# Synthesis of Aromatic Polyimides Based on 3,4′-Oxydianiline by One-Pot Polycondensation in Molten Benzoic Acid and Their Application as Membrane Materials for Pervaporation

**DOI:** 10.3390/ma15196845

**Published:** 2022-10-02

**Authors:** Anastasiia E. Soldatova, Regina N. Shamsutdinova, Tatiana V. Plisko, Katsiaryna S. Burts, Anna Yu. Tsegelskaya, Dmitry A. Khanin, Kristina Z. Monakhova, Tikhon S. Kurkin, Alexandr V. Bildyukevich, Alexander A. Kuznetsov

**Affiliations:** 1 Enikolopov Institute of Synthetic Polymer Materials, Russian Academy of Sciences, Profsoyuznaya St., 70, 117393 Moscow, Russia; 2 Institute of Physical Organic Chemistry, National Academy of Sciences of Belarus, Surganov St., 13, 220072 Minsk, Belarus; 3 Nesmeyanov Institute of Organoelement Compounds, Russian Academy of Sciences, Vavilova St., 28, bld. 1, 119334 Moscow, Russia

**Keywords:** polyimides, polycondensation, thermoplastic polymers, semi crystalline polymers, membrane, pervaporation

## Abstract

A series of aromatic polyimides based on the asymmetrical diamine 3,4ʹ-oxydianiline and various tetracarboxylic acid dianhydrides, both “rigid” and “flexible” structure, have been synthesized using the original method of one-pot high-temperature catalytic polycondensation in molten benzoic acid. The synthesized polyimides were investigated using fourier-transform infrared (FTIR) and ^1^H NMR spectroscopy, gel permeation chromatography (GPC), differential scanning calorimetry (DSC), thermogravimetric analysis (TGA), thermomechanical analysis (TMA) and wide-angle X-ray scattering (WAXS). It was found that the synthesized polyimides, depending on the used dianhydride, are characterized by different solubility in organic solvent and molten benzoic acid, molecular weight, glass transition temperature (Tg) from 198 to 270 °C, an amorphous or semi crystalline structure with the degree of crystallinity from 41 to 52%. The influence of the method of synthesis on the formation of the crystalline phase of polyimides was studied, and the obtained results were compared with the literature data. The effect of dianhydride chemical structure on the performance of polyimide in pervaporation more specifically, dehydratation of azeotropic isopropanol solution was investigated and compared with the commercially available polyetherimide Ultem 1000™. Membrane structure was studied using scanning electron microscopy. It was found that polyimide PI-DA is the most effective for separation of 88 wt.% isopropanol/12 wt.% water mixture compared to the polyimide PI-6FDA and commercial polyetherimide Ultem 1000™ demonstrating normalized permeation flux of 2.77 kg µm m^−2^ h^−1^ and separation factor of 264 (water content in permeate 97 wt.%).

## 1. Introduction

Aromatic polyimides (PIs) represent the class of polymers with unique properties due to their chemical structure [1,2]. They have high heat resistance, thermo-oxidative stability, radiation resistance and mechanical properties and can be used in extreme conditions. However, due to the rather rigid structure of repeating units, as well as intense intermolecular interactions, some aromatic PIs have poor solubility and processability [3,4].

It was shown in the previous reviews, that there are several ways to avoid these problems. One of possible approaches consists in the use of monomers with an asymmetric structure [3]. Asymmetry can be achieved both by introducing additional substituents in the monomer, and by the use of isomeric structures—the simplest option from a synthetic point of view. Moreover, such isomeric monomers are often commercially available. The introduction of such fragments diminishes of intermolecular interactions of polyimide macromolecules, but at the same time, a rigid structure can be preserved, which makes it possible to maintain high thermal properties.

The asymmetric diamine 3,4′-oxydianiline (3,4′-ODA) is often used in combination with dianhydrides with «rigid» structure to impart thermoplasticity and solubility to such polyimides characterized by high glass transition temperatures.

An example of such molecular design is the development of LARC™-IA (Langley Research Center-Improved Adhesive) polyimide synthesized from 3,4′-ODA and “semi-rigid” 4,4′-oxydiphthalic anhydride (ODPA) having a glass transition temperature of 220 °C [5]. It is thermoplastic polyimide; fibers, films, etc. are made from the melt of this polymer [6,7,8]. Also, the ability of the polyimide to form a crystalline phase is noted in the literature [6]. In turn, PI based on symmetric 4,4′-oxydianiline (4,4′-ODA) is amorphous with a glass transition temperature of 275 °C; this PI is obtained mainly in the form of films through the precursor—polyamic acid [9].

Commercially available highly heat-resistant PI Upilex S™ (Ube) [10] with a glass transition temperature above 400 °C is based on 3,3’,4,4’-biphenyltetracarboxylic acid dianhydride (BPDA) and symmetrical 4,4′-ODA which is available only in the form of films is designed. It was reported that the replacement of diamine with an asymmetric one leads to the formation of semi crystalline PI with temperatures glass transition and melting of 251 °C and 402 °C, respectively, capable of processing by extrusion and injection molding [11].

Copolyimide of amorphous morphology LARC™-SI is developed by Langley Research Center, SI means Soluble Imide. It is synthesized from an equimolar amount of dianhydrides ODPA, BPDA and 3,4′-ODA [12]. The LARC™-SI copolyimide contains two anhydride fragments of a sufficiently “rigid” structure, each of which, in combination with 3,4′-ODA, forms insoluble polymers [9,11]. Despite the fact, when copolymerized in this composition, these dianhydrides yield a polymer, soluble in high-boiling aprotic polar solvents. However, at the same time, LARC™-SI copolyimide loses solubility after heating it above Tg 240 °C.

The combination of 3,3′,4,4′-benzophenonetetracarboxylic dianhydride with symmetrical 4,4′-ODA results the formation of a partially crystalline polyimide with a Tg of 300 °C; no melting point up to 400 °C was observed, so, the obtained PI, just like the previous described “symmetrical” PI, is unsuitable for processing by extrusion and injection molding in the standard technological window for high-temperature polymers [13].

The morphology and degree of crystallinity of PIs is known to be strongly affected by the backbone chain flexibility, polydispersity index and molecular weight distribution, along with applied external conditions such as the presence of a suitable solvent, heat transfer balance and external mechanical load. From the technological perspective, the correlation between the properties and morphology of PIs and their method of preparation is of considerable interest. Analysis of the previously published results allows for the conclusion that the crystallization rate and degree of crystallinity in PIs can sometimes be successfully altered by tuning their preparation method. For example, one-stage high-temperature synthesis of PI in m-cresol based on 3,4′-ODA and BPDA with isolation of PI as a powdered product by precipitation with methyl ethyl ketone is described by Tamai et al. [11]. The degree of crystallinity of the thus obtained PI is 25%. When obtaining PI of the same structure, but by a two-stage method through polyamic acid and with further thermal imidization, as shown in [14], no crystalline phase is observed. Apparently, this effect is associated with the viscosity of the system in which the crystalline phase of the polymer is formed. It is generally considered that PI crystallization from solution, spontaneous precipitation from the reaction medium, or precipitation into another solvent is more favorable for the formation of a crystalline phase, than thermal imidization, due to the relatively low viscosity of the solution. When the thermal imidization is applied, the formation of a crystalline phase is hindered due to diffusion restrictions in addition to other factors that affect the crystallization rate. A similar effect of the preparation method on the degree of crystallinity of polyamidoimides was reported in [15].

In research of Koning, the influence of synthesis conditions on the properties of polyimides with aliphatic fragments crystallizing from the melt was studied, but from the point of view of possible side reactions [16]. In that work, PIs obtained by different methods were compared, and the effect of the presence of end groups in PI on crystallization was also investigated. End groups can participate in side reactions leading to the formation of nonlinear structures, which in turn hinder crystallization process.

In the present work, aromatic PIs based on 3,4′-ODA were synthesized by an original method of high-temperature catalytic polycondensation in a benzoic acid melt [17]. This method, in comparison with traditional ones, is more environmentally friendly, technologically simple; due to the strong catalytic effect of benzoic acid, the first stage (acylation reaction) kinetically disappears, and the two-stage process seems to be reduced to one stage [18]. Previously, it was shown that this method is effective for the synthesis, including polyimides of different topology (hyperbranched, star-shaped), block copolymers, as well as using low-reactive monomers [19,20,21,22].

In the present work, a series of aromatic PIs was synthesized based on asymmetrical 3,4′-ODA and dianhydrides of various structures, of both “rigid” and “flexible” fragments. The effect of the dianhydride structure on their thermal and mechanical properties, solubility, and crystallinity was studied, and the influence of the PI synthesis method on the formation of the crystalline phase was evaluated in comparison with literature data. In addition, PIs soluble in low-boiling solvents were studied as membrane material for separation of isopropyl alcohol-water mixture by vacuum pervaporation.

## 2. Materials and Methods

### 2.1. Materials

The monomers 3,4′-Oxydianiline (3,4′-ODA), 4,4′-(4,4′-Isopropylidenediphenoxy)bis(phthalic anhydride (DA), 4,4′-Oxydiphthalic anhydride (ODPA), 4,4′-(Hexafluoroisopropylidene)diphthalic anhydride (6FDA), 3,3′,4,4′-Biphenyltetracarboxylic dianhydride (BPDA), Benzophenone-3,3′,4,4′-tetracarboxylic dianhydride (BZP), were purchased from Sigma-Aldrich and were used without further purification. The 1,4-Bis(3,4-dicarboxyphenoxy)benzene dianhydride (RDA)) provided by Prof. I. G. Abramov (Yaroslavl State Technical University, Yaroslavl, Russia). Benzoic acid (BA) was crystallized from a water–acetone mixture and dried in vacuo (Tm = 122 °C).

### 2.2. Typical Synthesis of PI

Crystalline BA (9.0 g) and 0.7287 g (0.0014 mol) DA were loaded into a glass reactor equipped with a stirrer and an inert gas inlet. After inert gas was blown into the reactor, the mixture was heated up to 140 °C, and 0.2803 g (0.0014 mol) of 3,4′-ODA was added. The reaction mixture was stirred for 2 h at 150 °C with slow blowing of inert gas. Then, a hot viscous liquid reaction mixture was poured into a glass cup and cooled down to 25 °C. After cooling, the solidified reaction mixture was grounded and washed repeatedly with acetone or diethyl ether to extract BA. Polymer residue was filtered and dried in vacuo. The yield of PI was close to 100%.

Other PIs were synthesized similarly.

### 2.3. Preparation of PI films

The PI films were obtained by hot pressing of the PI powder using a laboratory press at 300–380 °C or from a 2% solution in chloroform. Films of PIs soluble in volatile solvents (Ultem 1000™(Riyadh, Saudi Arabia), PI-DA and PI-6FDA) for study of transport properties in the process of separation of isopropyl alcohol/water mixture by vacuum pervaporation were prepared using 1 wt.% solutions in chloroform. Commercial Ultem 1000™ was studied for comparison since its macromolecule contains a fragment the same dianhydride (4,4′-(4,4′-Isopropylidenediphenoxy)bis(phthalic anhydride) as PI-DA. Dissolution of PI in chloroform was held at room temperature using magnetic stirrer for 4 h. Then the solution was filtered using glass Shott Filter (pore size 40 µm). Calculated amount of the PI solution was poured on leveled Petri dish to obtain the films of the similar thickness and was left for evaporation in the fume hood for 5 h at T = 25 °C.

### 2.4. Methods

The Fourier-transform infrared (FTIR) spectra were measured using a Bruker Equinox 55/S spectrometer (Billerica, MA, USA). The samples were prepared in the form of a tablet with CsI pellet (the content of the test substance was 1%), and the films were obtained by pressing at temperatures above 300–380 °C.

^1^H NMR were registered on Bruker (Billerica, MA, USA) AM-300 spectrometer at 300.13 MHz. Chemical shifts are given with respect to signal of Si(CH_3_)_4_.

Gel permeation chromatography (GPC) measurements were executed on the device “Stayer” (Akvilon, Moscow, Russia) at 60 °C, with Phenogel column (2–70 kDa) and DMF as eluent (1 mL/min). Polystyrene standards were used.

Inherent viscosity of PI was measured in DMF at the concentration of 0.5 g/dL at 25 °C or mixture of p-chlorophenol/phenol/ (9:1) of 0.5 g/dL at 35 °C.

The study by differential scanning calorimetry (DSC) method was conducted on a DSC-3 device (Mettler-Toledo, Nänikon, Switzerland) at a heating rate of 10 °C/min in argon.

Thermogravimetric analysis (TGA) was performed with a Simultaneous Thermal Analysis device DTG-60H (Shimadzu, Kyoto, Japan) at a heating rate of 10 °C/min in air and argon.

Thermomechanical analysis (TMA) was performed with a thermomechanical analyzer TMA/SDTA 2+ Mettler-Toledo, Nänikon, Switzerland at a heating rate of 5 °C/min in penetration mode with constant load 0.5 N using a quartz stem with a ball-point tip, diameter 3 mm.

Wide-angle X-ray scattering (WAXS) data was obtained using a Bruker D8 Advance (Bruker AXS GmbH, Billerica, MA, USA) diffractometer equipped with X-Ray tube (λ(CuKα) = 1.5418 Å), Ge-crystal monochromator, Ni-filter and LYNXEYE detector. X-ray diffraction patterns were obtained in transmission mode (2θ = 10–50°), with powder samples placed between two layers of amorphous poly(ethylene terephthalate) (PET) films in a sample holder rotating at 90 RPM. Background scattering and scattering from amorphous PET were subtracted from the measured curves using DIFFRAC EVA and Origin 2020 Pro software. The degree crystallinity was calculated according to [23]. Is2-s plots were used to calculate the X-ray scattering intensities of the ordered (presumably, crystalline) and disordered (amorphous) polymer phases, where I is the scattering intensity and s = 2 sin(θ)/λ is a reciprocal space vector modulus.

Mechanical studies of the PIs were performed using universal electromechanical test machine Hounsfield H1KS (Redhill, UK). The size of each sample stretched area was 15 × 3 mm^2^, the mat’s thickness was in the range from 80 μm to 110 μm. All the tensile tests were carried out at an elongation rate of 5 mm/min. Experiments of each type were carried out at least three times.

Structure of PI membranes (cross-section and surface) were investigated using Phenom Pro scanning electron microscope (Thermofisher Scientific, Waltham, MA, USA). Membranes were fractured in liquid nitrogen and then the samples were coated by a gold layer of 1 nm using vacuum sputter coater DSR (Vaccoat, London, UK).

Membrane water contact angles were determined by the sessile drop technique using LK-1 goniometer (Otktrytaya Nauka, Krasnodar, Russia). Five different samples were measured of and average value was calculated. A measurement error was lower than ±2°.

### 2.5. Study of PI Films Performance in Vacuum Pervaporation

Transport properties of PI films were investigated by vacuum pervaporation of isopropanol/water mixtures with 12 wt.% of water in the feed solution at feed solution temperature of 25 °C. The pressure from permeate side was lower than 0.01 mmHg. The scheme of pervaporation set-up is described in [24]. The feed and permeate compositions were analyzed by Chromatec Crystal 5000.2 gas chromatograph (Chromatec, Yoshkar-Ola, Russia). Membrane permeation flux (J, kg m^−2^ h^−1^) was calculated by Equation (1):

(1) $$J=\frac{m}{S\cdot t}$$

where m is the permeate weight, g; S is the effective membrane area, m^2^; t is the time of pervaporation, h.

Moreover, the normalized flux (J_N_, kg µm m^−2^ h^−1^) was calculated considering membrane thickness (L) by Equation (2):

J_N_ = J·L, (2)

L was determined using SEM micrographs of PI films.

Pervaporation separation index (PSI, kg m^−2^ h^−1^) was calculated by Equations (3) and (4):

(3) β=yAyBxAxB,

PSI = J·(β − 1),(4)

where β is separation factor; J is permeation flux, kg m^−2^ h^−1^; y_A_ and y_B_ are water and isopropyl alcohol concentrations in the permeate, respectively; x_A_ and x_B_ are water and isopropyl alcohol concentration in the feed solution, respectively.

Moreover, the thickness normalized pervaporation separation index (PSI_N_, kg µm m^−2^ h^−1^) was determined by Equation (5). PSI_N_ is a factor used to compare the pervaporation performance of various membranes in the same pervaporation process [25].

(5) $${\mathrm{PSI}}_{N}=J_{N}\left(\beta -1\right),$$

where J_N_—normalized permeation flux, kg µm m^−2^ h^−1^.

The dependence of membrane flux on temperature is described by the Arrhenius relation (Equation (6)):

(6) $$J_{x}=J_{x,0}\exp\left(-\frac{E_{\mathrm{app}}}{\mathrm{RT}}\right),$$

where J_x_ is the partial flux of components (ethanol and water) (g m^−2^ h^−1^), J_x,0_ is the pre-exponential factor, E_app_ is the apparent activation energy (J mol^−1^), R is the gas constant (J mol^−1^ K^−1^), T is the absolute temperature (K).

To determine E_app_ the partial flux of components was determined in pervaporation of the mixture 88 wt.% isopropanol/12 wt.% water at 25 °C, 35 °C and 50 °C. E_app_ was calculated from the slope of the plot of the dependence of lnJ_x_ on 1000/T according to the Equation (6).

## 3. Results

### 3.1. Synthesis and Chemical Structure

**Scheme 1:**
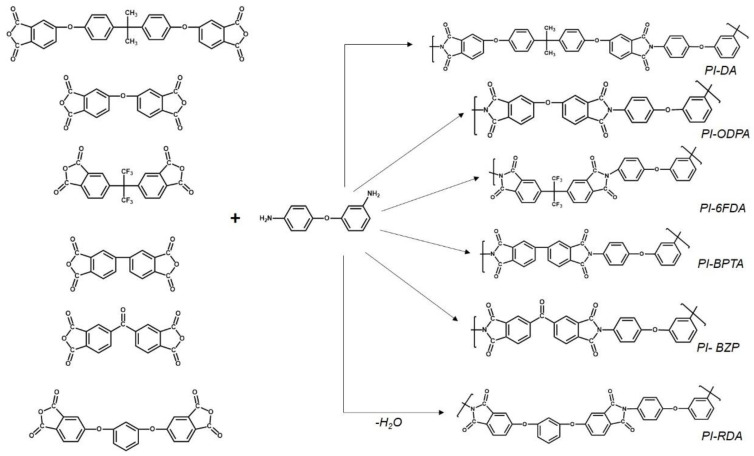
The scheme of synthesis and the chemical structure of the PIs.

Aromatic polyimides based on unsymmetrical diamine 3,4′-ODA and various tetracarboxylic acid dianhydrides, both with rigid and flexible fragments were synthesized via the method of polycondensation in the molten BA (Scheme 1).

It was found that depending on the selected dianhydride, the polycondensation products have different solubility in the molten benzoic acid. PI samples PI-ODPA, PI-BPDA and PI-BZP based on «rigid» and «semi-rigid» dianhydrides fall out of the molten BA in the form of powders within 5–10 min from the start of the process.

Table 1 shows the data of the solubility of the obtained PI. PI samples based on dianhydrides with «flexible» fragments or fluorinated fragment PI-DA and PI-RDA and PI-6FDA demonstrated good solubility in different types of solvents (BA, aprotic solvents including amide aprotic solvents, halogenated hydrocarbon, cyclic ether). Samples PI-DA and PI-6FDA are soluble in low boiling solvents such as THF and chloroform. It was revealed that PIs composed of dianhydrides of a «rigid» and «semi-rigid» structure, do not dissolve in organic solvents.

**Figure 1:**
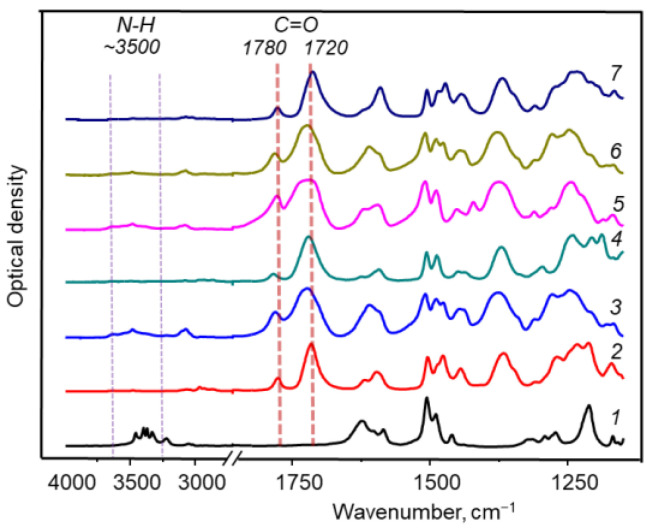
IR spectra of 3,4′-ODA (1) and polyimides PI-DA (2), PI-ODPA (3), PI-6FDA (4), PI-BPTA (5), PI-BZP (6) and PI-RDA (7).

The IR spectra of the initial diamine 3,4′-ODA and the aromatic PIs derived from it are presented in Figure 1. When comparing the IR spectra of diamine and polyimides, the spectra of the reaction products show the appearance of absorption bands in the region of 1720 cm^−1^ and 1780 cm^−1^, corresponding to symmetric and asymmetric stretching of the C=O bond in the imide cycle. Absorption bands disappear in the region of 3250–3500 cm^−1^, which is related to stretching of the N-H bond of the amino group of initial 3,4′-ODA. However, in the spectra of samples of PI-ODPA, PI-BPTA, PI-BZP peaks of low intensity at ~3500 cm^−1^ are observed, which can be attributed to absorption bands also N-H bond of the end amino groups of the polycondensation product. In this case, due to the rigid structure or tendencies to ordering of the polycondensation product, they precipitate from the reaction mixture in the form of a powdered product, which can be suggested as an oligomer with terminal functional groups. 

**Figure 2:**
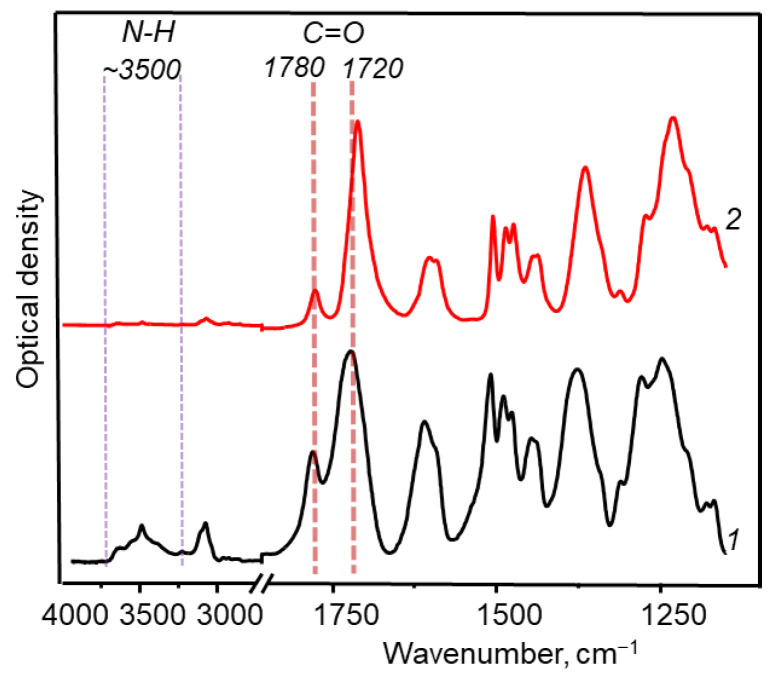
IR spectra of polyimides PI-ODPA before (1) thermal treatment and after (2).

Also, during thermal treatment above the melting point of the powdered product of PI-ODPA, PI-BPTA, PI-BZP, conditions are created for the reaction between the end groups to proceed and a high molecular weight product is formed. Comparison of the IR spectra of the powder and the film shows that there is no N–H bond vibrations in the region of 3500 cm^−1^ in the film spectrum, also changes are observed in the absorption bands 1720–1780 cm^−1^. For example, the spectrum of PI-ODPA is shown on Figure 2. This assumption is also confirmed by solution viscosity measurements given below (Table 2).

**Figure 3:**
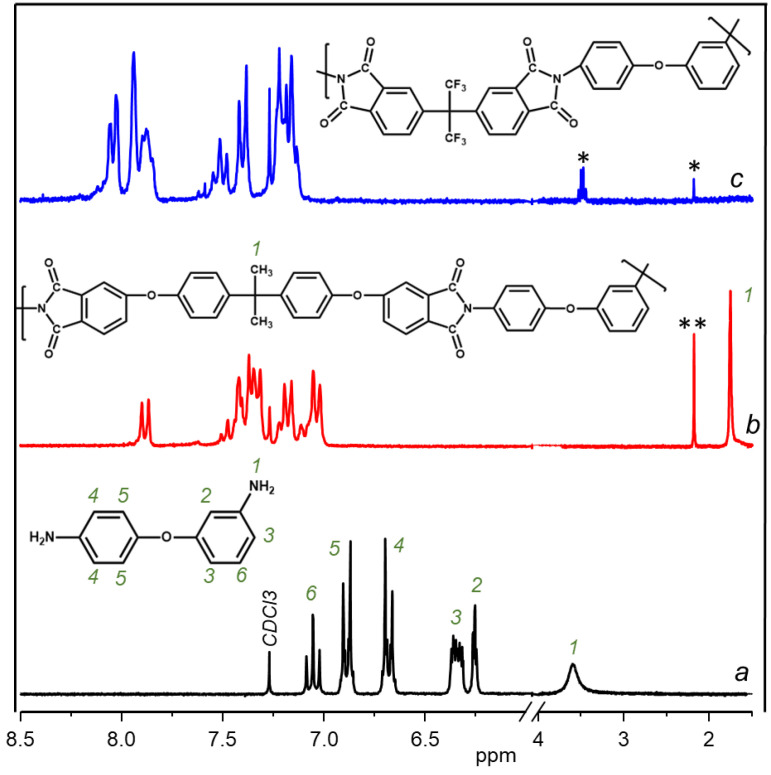
^1^H NMR spectra of 3,4′-ODA (a), PI-DA (b) and PI-6FDA (c) in CDCl_3_ (residual solvents: *—diethyl ether, **—acetone).

Figure 3 shows ^1^H NMR spectra of the initial diamine 3,4′-ODA and soluble polyimides PI-DA and PI-6FDA samples. The samples of PI-ODPA, PI-BPTA, PI-BZP are insoluble in organic solvents (including CDCl_3_ and DMSO-*d_6_*) and therefore weren’t analyzed by this method. When comparing the spectra of the initial diamine and polyimides PI-DA and PI-6FDA, the spectra of the polycondensation products do not contain signals in the region of 3.6 ppm, which correspond to the proton signals of the amino group of the diamine. Aromatic signals assigned to 3,4′-ODA fragments after polycondensation with dianhydrides are shifted towards weak fields. In the spectrum of PI-DA (b), signals are observed in the high field region of 1.76 ppm, related to the aliphatic protons of the dianhydride A fragment. In the spectrum of the PI-6FDA sample, intense signals of aromatic protons are observed, they are divided into two groups, related to the 6FDA fragment and to diamine protons.

### 3.2. Molecular Weight Characteristics and Mechanical Properties of Soluble PIs

Molecular weight characteristics of soluble polyimides were determined by GPC with calibration according to PS standards and viscometry in DMF solvent. Samples are characterized by high molecular weights (Table 2).

To estimate the molecular weights of PIs insoluble in organic solvents by viscometry, a non-trivial solvent mixture of p-chlorophenol/phenol (9:1) was used. The viscosity of both powder products from the synthesis and films obtained by pressing them in a high-temperature press was measured. It was assumed that during thermal pressing reactions occur between the terminal amino and anhydride groups of the polycondensation products and an increase in molecular weights occurs. This assumption is confirmed by increasing values of the viscosity of solutions of powdered products and films, for example, a solution of PI-BPDA powder isolated from the synthesis has a viscosity of 0.71 dL/g, after pressing 1.28 dL/g (Table 2).

**Figure 4:**
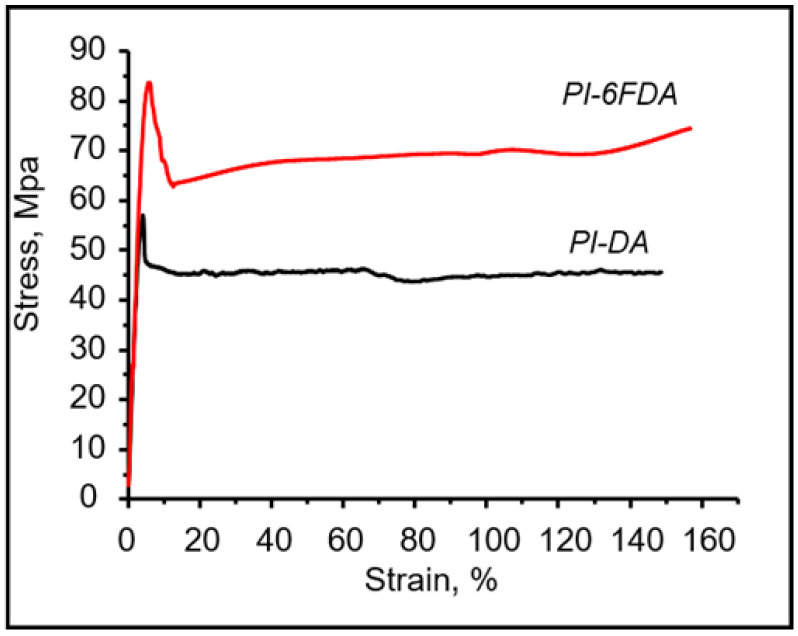
Typical stress-strain curves at room temperature of film cast from chloroform PI-DA and PI-6FDA (elongation rate of 5 mm/min).

The mechanical properties of films obtained from chloroform solutions of PI-DA and PI-6FDA in the stretching mode at room temperature were studied (Figure 4). It was found that the samples deform to form a neck with a tensile strength of 67.4 ± 10 and 82.8 ± 5 MPa, an elongation at break of 137 ± 23% and 147 ± 17% and an elastic modulus of 1.9 ± 0.17 and 2.7 ± 0.17 GPa for samples PI-DA and PI-6FDA, respectively.

### 3.3. Thermal Properties

**Figure 5:**
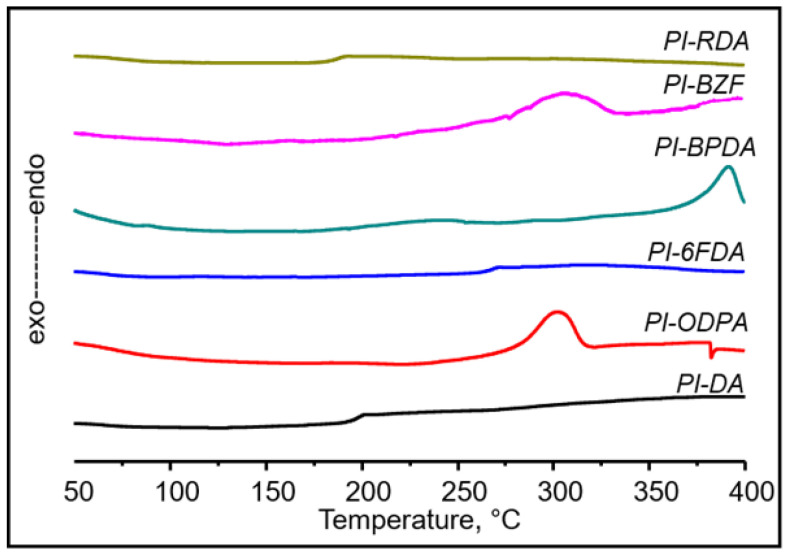
DSC curves of obtained PI. The first scanning. (in argon atmosphere, 10 °C/min).

Thermal properties of polyimide samples were analyzed by methods DSC, TGA and TMA. DSC curves (first scanning) of polycondensation products are shown in Figure 5. On the curves of the samples PI-ODPA, PI-BPDA and PI-BZP endothermal peaks at the temperature 302, 390 and 306 °C are observed, respectively.

**Figure 6:**
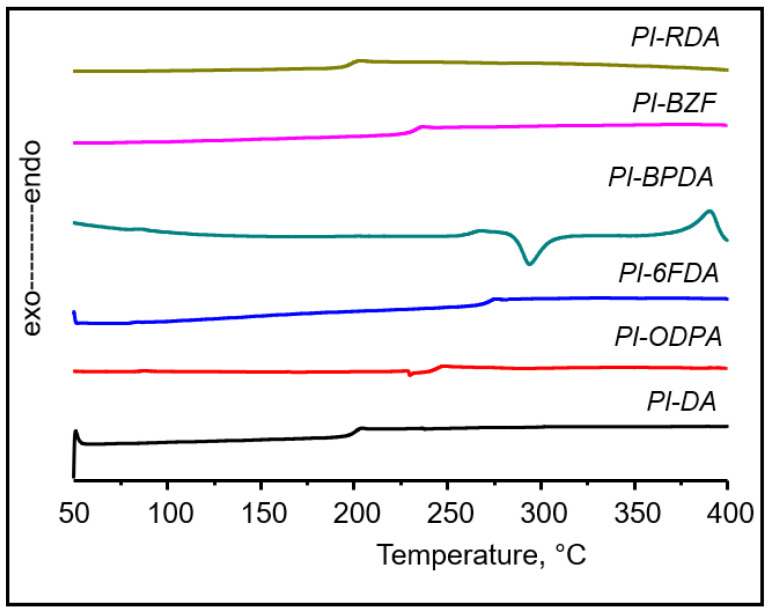
DSC curves of obtained PI. The second scanning. (in argon atmosphere, 10 °C/min).

The endothermal peaks on the DSC curves indicate a presence of a crystalline phase in these samples. However, during the second scan (Figure 6), the crystalline phase was registered only in the sample PI-BPDA, based on 3,3′,4,4′-biphenyltetracarboxylic acid dianhydride. It can be noted that the PI sample with BPDA fragments shows a strong tendency to form a crystalline phase.

It is also worth to note that the glass transition temperatures during the first scan of product of polycondensation were recorded only for samples PI-DA, PI-6FDA and PI-RDA. Basically, this shows the ratio between the amorphous and crystalline phases in the samples PI-ODPA, PI-BPDA and PI-BZP, obviously, the amorphous phase is not large and therefore no glass transition temperature is observed on DSC during the first scan. On the DSC thermograms (second scan), the glass transition temperature is observed for all samples (Figure 6).

**Figure 7:**
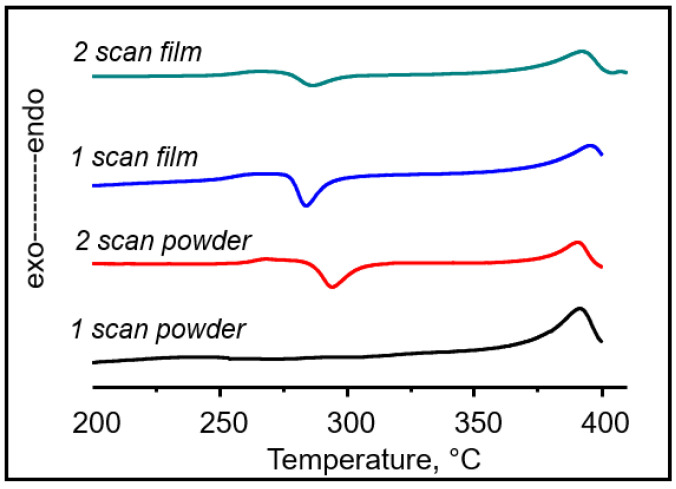
DSC curves of PI-BPDA (powder and film) (in argon atmosphere, 10 °C/min).

Also, PI-BPDA film demonstrated the presence of crystalline phase. On DSC thermogram, peaks of «cold crystallization» and melting were observed at the first and second scans (Figure 7). Table 3 shows DSC crystallization data for sample PI-BPDA. The heat of melting of the film after the second scan (∆*H_m_* = 17.77 J/g) increases compared to the first scan (∆*H_m_* = 5.81 J/g), which confirms the fact that the polymer of such a structure has a strong tendency to crystallize and the crystalline phase is not only preserved, but when certain conditions are created (slow cooling (20 °C/min)-heating (10 °C/min) is conditions DSC) the amount of the crystalline phase increases.

In general, concerning glass transition temperature, there is a direct relationship between the structure of the selected dianhydride and the glass transition temperature: the more «rigid» the structure of dianhydride gives the higher the glass transition temperature of the polyimide.

**Figure 8:**
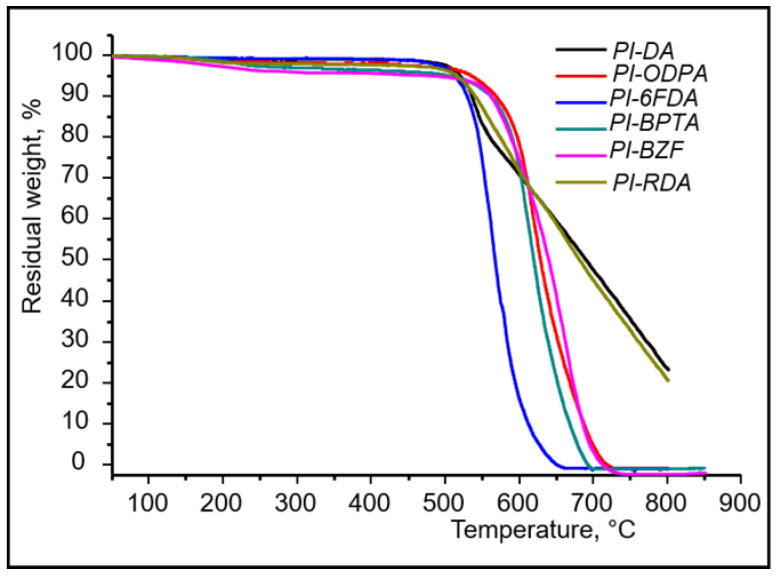
TGA curves of polyimides powder (in air atmosphere, 10 °C/min).

Before discussing the thermo-oxidative stability data, it should be remembered that PI-ODPA, PI-BPDA and PI-BZP samples are oligomers with end groups, the presence of which can reduce thermal stability, especially in air. Indeed, when comparing the thermal-oxidative stability of products isolated from the synthesis (powder) and after heat treatment (film), an increase in temperature of thermo-oxidative degradation with 5% weight loss (T_d_^5%^) set up is observed (Table 4). It is also interesting to note different character of thermal-oxidative degradation for samples PI-DA and PI-RDA (Figure 8) compared to other PIs. The char yields in air at temperature of 800 °C for PI-DA and PI-RDA were 24% and 21%, respectively, while for all the other PIs it was 0%.

**Figure 9:**
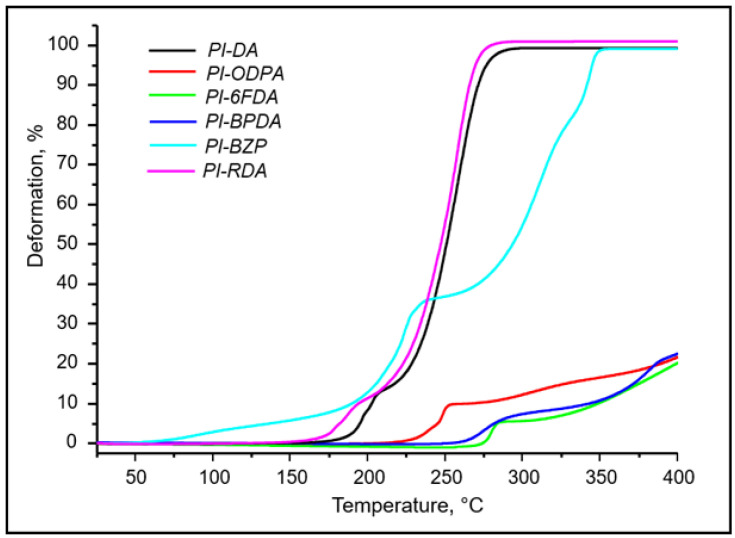
TMA curves of synthesized polyimides (heating rate of 5 °C/min in penetration mode with constant load 0.5 N).

The results of thermomechanical analysis at a constant load (0.5N) in the penetration mode for films prepared in thermal press are shown in Figure 9. On the TMA curves of all samples, deformation is observed, associated with the transition from the glass state to the elastic state. On the curves of the samples with flexible fragments PI-DA, PI-RDA and PI-BZP, a clear transition from the high-elastic to the state of viscous flow is observed, while in the PI-BZP sample, complete deformation is observed only when the melting temperature is reached. Samples based on dianhydrides with rigid fragments PI-ODPA, PI-BPDA and PI-6FDA do not show a transition from an elastic to a flow state under the conditions of analysis.

### 3.4. Morphology of Obtained Polyimides

In this work, the WAXS method was used to study the phase morphology of the synthesized PIs; on the DSC thermograms, which show endothermic peaks, PI-ODPA, PI-BPDA, and PI-BZP, powdered products isolated from the synthesis were analyzed.

**Figure 10:**
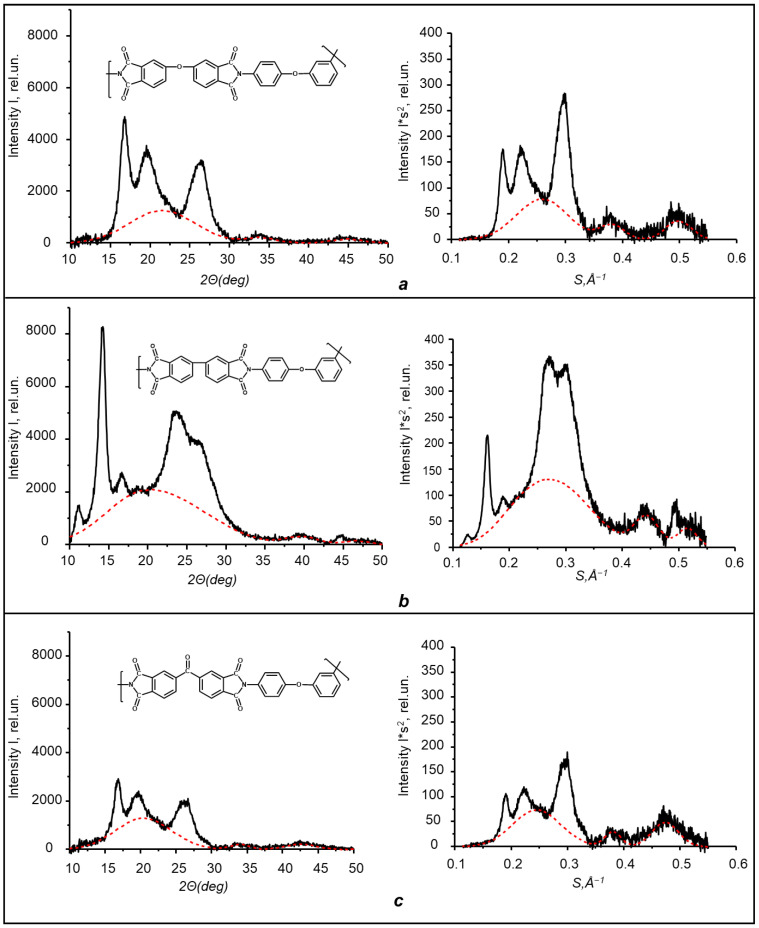
The WAXS diffractograms of PI-ODPA (**a**), PI-BPDA (**b**) и PI-BZP (**c**). Experimental WAXS curves are black. Calculated scattering curves from amorphous (disordered) phase are red.

**Figure 11:**
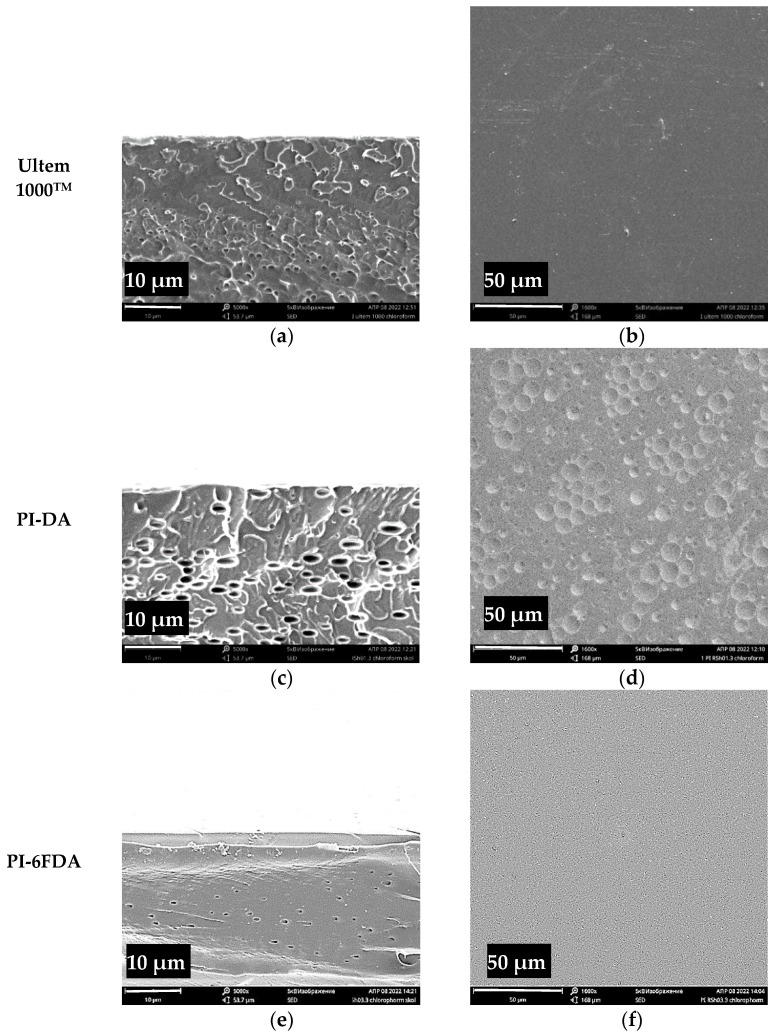
SEM images of the cross-section (**a**,**c**,**e**) and surface of the selective layer (**b**,**d**,**f**) of membranes based on Ultem 1000™, PI-DA and PI-6FDA.

Figure 10 shows WAXS background-corrected scattering curves of PI-ODPA, PI-BPDA and PI-BZP samples plotted in I-2Ɵ coordinates (left) and Is2-s coordinates (right). The obtained WAXS scattering data indicates that the PI-ODPA, PI-BPDA, and PI-BZP samples contain partially ordered phase, so it can be assumed that these polymers are semi-crystalline. The degree of crystallinity of the PI-ODPA, PI-BPDA, and PI-BZP samples, determined from the WAXS scattering curves in Figure 11 (left) was 52, 44 and 41%, respectively.

Comparing these results with the literature data leads to a conclusion that the process conditions during polycondensation in BA are more favorable for obtaining PIs with a high degree of crystallinity. Whereas, for example, a sample based on 3,4′-ODA and 3,3′,4,4′-biphenyltetracarboxylic acid dianhydride, obtained by one-stage high-temperature polycondensation in m-cresol and isolated by precipitation into methyl ethyl ketone, is characterized by a degree of crystallinity of 25% [10].

### 3.5. Study of Transport Properties PIs in Pervaporation

Pervaporation can be an efficient tool to evaluate structure of the polymer since performance in pervaporation separation depends on chemical nature of membrane polymer, membrane structure (cavity size, thickness), degree of crystallinity, fractional free volume of polymer and interaction of the components of the feed mixture with membrane material and between each other. It is generally accepted that transport in pervaporation takes place in a three-step mechanism which can be described by solution-diffusion model: (i) sorption into the membrane, (ii) diffusion through the membrane, and (iii) desorption into the vapor phase [26]. According to solution-diffusion model the permeability of a component depends both on membrane sorption coefficient and the diffusion coefficient (mobility). PIs are interesting polymers for pervaporation studies because their structure can be manipulated in a broad range by combination of different dianhydrides and diamines [25]. PIs were found to be selective toward water due to the preferential interaction between water molecules and the imide groups by formation of hydrogen bonds. PIs were reported to feature diffusivity-controlled transport mechanism in separation of alcohol-water mixture via pervaporation [27,28].

In this study for preparation of dense pervaporation membranes PI-DA and PI-6FDA which are soluble in volatile solvents were selected (Table 1). This requirement is due to the fact that pervaporation membranes with non-porous selective layer are usually prepared via evaporation induced phase separation technique [29]. For comparison commercially available polyetherimide Ultem 1000™ based on 4,4′-(4,4′-isopropylidenediphenoxy)bis(phthalic anhydride) and m-phenylene diamine was used for preparation of pervaporation membranes (Figure 11).

Micrographs of the membrane cross-section and surface obtained by SEM are presented in Figure 11. It is worth noting that membranes prepared from Ultem 1000™ and PI-DA feature non-uniform structure with isolated blind pores in the cross-section. However, size of the pores is bigger for PI-DA membrane (2–3 μm) compared to pores for Ultem 1000™ (0.3–1 μm). Cross-section of the PI-6FDA membrane is more uniform and size of the isolated pores in the cross-section is smaller (0.5–1 μm) compared to the PI-DA membranes (Figure 11). It is worth noting that membrane surface of PI-DA is characterized by the presence of the craters unlike the smooth and uniform surface of Ultem 1000™ and PI-6FDA. Membrane transport properties in separation of 88 wt.% isopropyl alcohol/12 wt.% water azeotropic mixture via vacuum pervaporation are presented in Table 5.

It was found that PI-DA membrane is characterized by the highest permeate flux (J), thickness normalized flux (J_N_) and water content in permeate compared to Ultem 1000™ and PI-6FDA membrane. PI-FDA membrane feature the lowest permeate flux, thickness normalized flux and water content in permeate compared to Ultem 1000™ and PI-DA which yields the lowest separation factor (β) and thickness normalized pervaporation separation index (PSI_N_). The lowest flux of PI-6FDA membrane is due to the denser and uniform structure of the membrane and smaller size of isolated pores compared to other studied membranes (Figure 11). Moreover, PI-6FDA features the highest molecular weight (Table 2) and higher chain rigidity (Table 4) which may result in higher packing density of macromolecular chains and decrease of fractional free volume of polymer which decreases the diffusion rate of penetrants during pervaporation. Low water content in permeate and low separation factor are attributed to the increase of isopropyl alcohol sorption when fluorinated groups are introduced to the polyimide structure. This tendency was reported and studied in detail in [30]. Moreover, PI-6FDA membrane was shown to be more hydrophobic according to the water contact angle measurements compared to Ultem 1000™ and PI-DA membranes which proves its lower affinity to water (Table 5).

The highest flux and thickness normalized flux of PI-DA membrane compared to other studied membrane are attributed to the peculiarities of membrane morphology, chemical nature and physical properties of PI-DA. From the point of view of membrane structure higher membrane permeability is due to the (i) more porous structure of membrane cross-section which increases rate of diffusion of the components of the feed mixture and (ii) higher membrane surface area with craters which leads to the increase of sorption area for the components of the feed mixture. It is worth noting that such a membrane structure is determined by the peculiarities of phase separation of PI-DA during membrane preparation. Moreover, PI-DA features lower molecular weight (Table 2) and lower chain rigidity (Table 4) compared to PI-6FDA which may result in lower packing density of macromolecular chains. It is commonly known that lower packing density and lower degree of crystallinity increases the diffusion rate of penetrants in pervaporation separation. Presence of oxygen ether atom both in DA and 3,4′-ODA yields the highest affinity to water for PI-DA compared to PI-6FDA and Ultem 1000™ which increases membrane sorption selectivity and leads to the highest separation factor (Table 5).

Apparent activation energy of water and isopropanol for PI-DA and PI-6FDA membranes was calculated according to Equation (6) from the dependence of component flux on feed solution temperature (Table 6). It was found that the apparent activation energy of water for both PI-DA and PI-6FDA membranes was lower compared to the apparent activation energy of isopropanol due to the smaller water molecules size and higher affinity of water to PIs. Therefore, water predominantly penetrates through the membrane selective layer. Moreover, higher apparent activation energy of isopropanol demonstrates that isopropanol flux is more dependent on temperature changes. It is worth noting that apparent activation energy for water is much higher (~7 times) for PI-6FDA membrane which confirms that PI-DA features stronger affinity to water compared to PI-6FDA. It is worth mentioning that the relative difference of apparent activation energy of water and isopropanol is higher for PI-DA membrane compared to the PI-6FDA membrane which is due to higher selectivity toward water of PI-DA.

**Figure 12:**
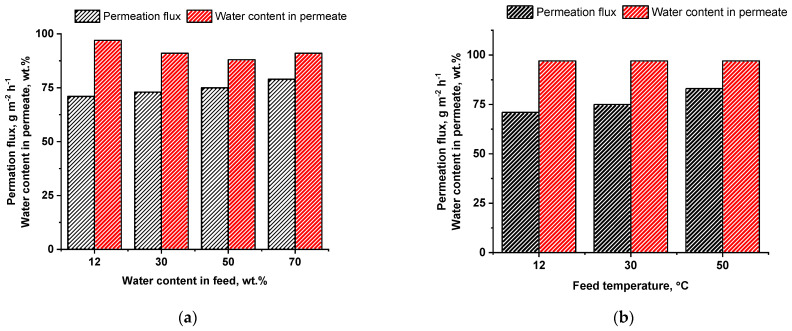

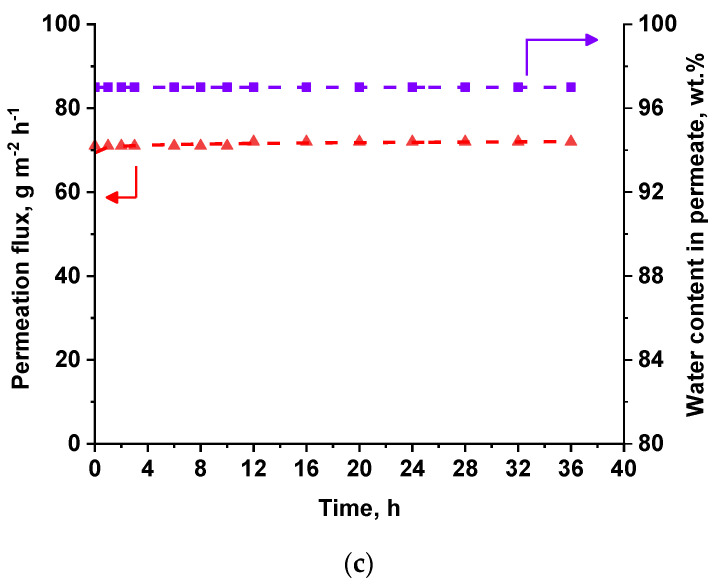
Pervaporation separation performance of PI-DA membrane in dehydrating of isopropanol: (**a**) dependence of permeation flux and water content in permeate on water content in the feed solution isopropanol/water (feed temperature is 25 °C); (**b**) dependence of permeation flux and water content in permeate on feed solution temperature (feed solution 88 wt.% isopropanol/12 wt.% water); (**c**) dependence of permeation flux and water content in the feed solution on time of pervaporation.

Thus, PI-DA is the most effective polyimide for separation of isopropyl-alcohol water mixture compared to the PI-6FDA and commercial Ultem 1000™. Therefore, PI-DA membrane was further investigated in pervaporation experiments (Figure 12).

It was found that with the increase of the water content in the feed solution the permeation flux of PI-DA membrane slightly increases and water content in the feed slightly decreases (Figure 12a). When water content in the feed solution increases from 12 wt.% to 70 wt.% the permeation flux increases from 71 to 79 g m^−2^ h^−1^ and water content in the permeate decreases form 97 to 88–91 wt.% (Figure 12a). It demonstrates that PI-DA membrane does not swell in dilute isopropanol solutions. Slight increase of permeation flux with the increase of water content in the feed is due to the increased water sorption from diluted aqueous isopropanol solutions. It was found that PI-DA membrane demonstrates low sensitivity of membrane pervaporation performance to the increase of feed solution temperature (Figure 12b). When the temperature of feed solution (88 wt.% isopropanol/12 wt.% water) increases from 25 °C to 50 °C permeation flux increases from 71 to 83 g m^−2^ h^−1^ and water content in permeate does not change (97 wt.%). Overall, the increase of permeation flux with the increase in feed temperature is attributed to the increase in vapor pressure (and chemical potential) which leads to the increase in driving force in pervaporation.

It was found that increase of PI-DA membrane thickness results in decrease of membrane permeation flux, however, thickness normalized permeation flux and separation factor don’t change (Table 5).

It was found that PI-DA membrane demonstrates constant flux and water content in permeate in pervaporation of 88 wt.% isopropanol/12 wt.% water mixture for 36 h which confirms its stability and efficiency in pervaporation of isopropanol-water mixtures with high isopropanol content (>88 wt.%) (Figure 12c). The absence of membrane swelling in the components of the feed mixture is due to the thermodynamic and kinetic rigidity of PI-DA macromolecule chain backbone which prevents macromolecular chain mobility.

It is worth noting that developed PI-DA membrane demonstrates competitive performance compared to data reported in the literature combining relatively high separation factor and flux [28,30,31,32,33,34,35,36,37,38]. Moreover, the excellent long-term pervaporation stability makes the developed PI-DA membrane promising material for isopropanol dehydration.

## 4. Conclusions

One-pot high-temperature polycondensation was used to synthesize a series of aromatic PIs based on asymmetrical diamine 3,4′-ODA and various dianhydrides.

Depending on structure the dianhydride used, PIs obtained are characterized by different solubility in organic solvents, including benzoic acid, different molecular weight, glass transition temperature from 198 to 270 °C, amorphous or crystalline morphology.

The present study shows that the use of the one-pot high-temperature polycondensation method leads to the production of semi crystalline PIs with a more developed crystalline phase, compared to traditional methods for the synthesis of polyimides. According to the temperature characteristics (T_g_ and T_m_) of the obtained PIs, as well as the character of the thermomechanical curve, it can be concluded that the synthesized PIs are processable by extrusion and injection molding. This issue will be the subject of further research. The synthesized PIs soluble in volatile solvents were studied as dense non-porous membranes for the separation of water-isopropyl alcohol azeotropic mixtures by vacuum pervaporation. It was found that PI based on 4,4′-(4,4′-isopropylidenediphenoxy)bis(phthalic anhydride) and 3,4′-ODA is the most effective PI for separation of isopropyl-alcohol water mixture compared to the PI based on 4,4′-(hexafluoroisopropylidene)diphthalic anhydride and commercial polyetherimide Ultem 1000™.

## Figures and Tables

**Table 1:** Abbreviation of synthesized PIs and their solubility.

Sample	Dianhydride	Solubility					
		BA	THF	CHCl_3_	NMP	DMAA	DMSO
PI-DA	4,4′-(4,4′-Isopropylidenediphenoxy)bis(phthalic anhydride)	+	+	+	+	+	+ *
PI-ODPA	4,4′-Oxydiphthalic anhydride	-	-	-	-	-	-
PI-6FDA	4,4′-(Hexafluoroisopropylidene) diphthalic anhydride	+	+	+	+	+	+
PI-BPTA	3,3′,4,4′-Biphenyltetracarboxylic dianhydride	-	-	-	-	-	-
PI-BZP	Benzophenone-3,3′,4,4′-tetracarboxylic dianhydride	-	-	-	-	-	-
PI-RDA	1,4-Bis(3,4-Dicarboxyphenoxy)Benzene dianhydride	+	-	+-	+	+	+ *

Solvent: BA—benzoic acid, THF—tetrahydrofuran, CHCl_3_—Chloroform, NMP—N-Methyl-2-pyrrolidone, DMAA—dimethylacetamide, DMSO—dimethyl sulfoxide. *—at 80 °C

**Table 2:** Molecular weight characteristics of obtained PI.

Sample	M_n_	M_w_	M_w_/M_n_	η, dL/g
PI-DA	44300	78200	1.8	0.39
PI-ODPA	-	-	-	0.86/ND *
PI-6FDA	108200	118500	1.1	1.04
PI-BPDA	-	-	-	0.71/1.28 *
PI-BZP	-	-	-	0.55/0.66 *
PI-RDA	46700	87300	1.9	0.39

M_n_—number average molecular weight; M_w_—weight average molecular weight; M_w_/M_n_—polydispersity index; η—inherent viscosity; * measured in p-chlorophenol/phenol mixture (9:1); before slash PI from the synthesis, after film obtained by pressing them in a high-temperature press; ND—no data, does not dissolve.

**Table 3:** DSC crystallization data for sample PI-BPDA.

DSC-Mode	State	*T_c_*_,_ °C	∆*H_c_*, J/g	*T_m_*, °C	∆*H_m_*, J/g
1 scan	powder	-	-	388	9.88
2 scan		294	13.77	390	14.21
1 scan	film	284	14.6	395	5.81
2 scan		286	7.53	392	17.77

T*_c_—*Temperature of crystallization; ∆*H_c_*—Heat of crystallization; *T_m_*—Melting point; ∆*H_m_*—Heat of melting.

**Table 4:** Thermal properties of obtained PIs.

Name of Sample	T_g_, °C			T_m_, °C			T_d_^5%^_,_ °C			
	1 Scan	2 Scan	Film	1 Scan	2 Scan	Film	Powder		Film	
							Air	Argon	Air	Argon
PI-DA	197	200	200	-			517	522	520	520
PI-ODPA	ND	244	244	302	-	-	535	570	551	554
PI-6FDA	268	270	270	-			512	530	525	515
PI-BPTA	ND	264	262	390	390	390	494	573	548	560
PI-BZP	ND	230	233	306	-	-	443	565	512	520
PI-RDA	187	198	198	-			515	522	511	520

*T_g_—*Glass transition temperature; *T_m_*—Melting point; *T_d_^5%^*—Temperature of thermal -oxidative degradation (5% weight loss).

**Table 5:** Transport properties of PI membranes in pervaporation of isopropyl alcohol/water mixture.

PI	J ^1^, kg m^−2^ h^−1^	L ^2^, µm	J_N_^3^, kg µm m^−2^ h^−^^1^	Water Content in Permeate, wt.%	β ^4^	PSI_N_^5^, kg µm m^−2^ h^−1^	Water Contact Angle, °
Ultem 1000™	0.032	36	1.15	74	21	0.63	87
PI-DA	0.071	39	2.77	97	264	18.65	87
PI-DA	0.045	61	2.77	97	264	18.65	87
PI-6FDA	0.015	28	0.42	62	12	0.17	92

^1^—permeation flux; ^2^—membrane thickness; ^3^—thickness normalized permeation flux; ^4^—separation factor; ^5^—thickness normalized pervaporation separation index.

**Table 6:** The apparent activation energy of water and isopropanol of PI-DA and PI-6FDA membranes.

Membrane Designation	E_app_ (Water), J mol^−1^	E_app_ (Ethanol), J mol^−1^
PI-DA	6.08	14.35
PI-6FDA	42.26	52.66

## Data Availability

The data presented in this study are available on request from the corresponding author. The data are not publicly available due to being a part of ongoing research.
